# Medical Journal on a New Plan

**Published:** 1833

**Authors:** 


### III. Medical Journal on a New Plan.

We have received the first number of a new periodical, entitled *The Register and Library of Medical and Chirurgical Science, a medical newspaper, edited by* Granville Sharp Pattison, M. D., Professor of Anatomy in Jefferson Medical College, Philadelphia. It is a single sheet of 64 octavo pages! The reason for using such a sheet, is the saving of postage to the subscribers. One part of this newspaper is devoted to original and analectic intelligence, the other to the republication of standard works in the profession, which may be bound up in separate volumes. The present number contains 48 pages of Sir Charles Bell's Treatise on the Nervous System, a work which we need hardly say, should be in the hands of every physician. The high professional reputation of Professor Pattison, and the cheapness of his Register, should recommend it to extensive patronage.

